# Supplementary figures and images for: Connexin hemichannels with prostaglandin release in anabolic function of bone to mechanical loading
Source: eLife. 2022 Feb 8;11:e74365. doi: 10.7554/eLife.74365 (PMC8824479; doi:10.7554/eLife.74365)

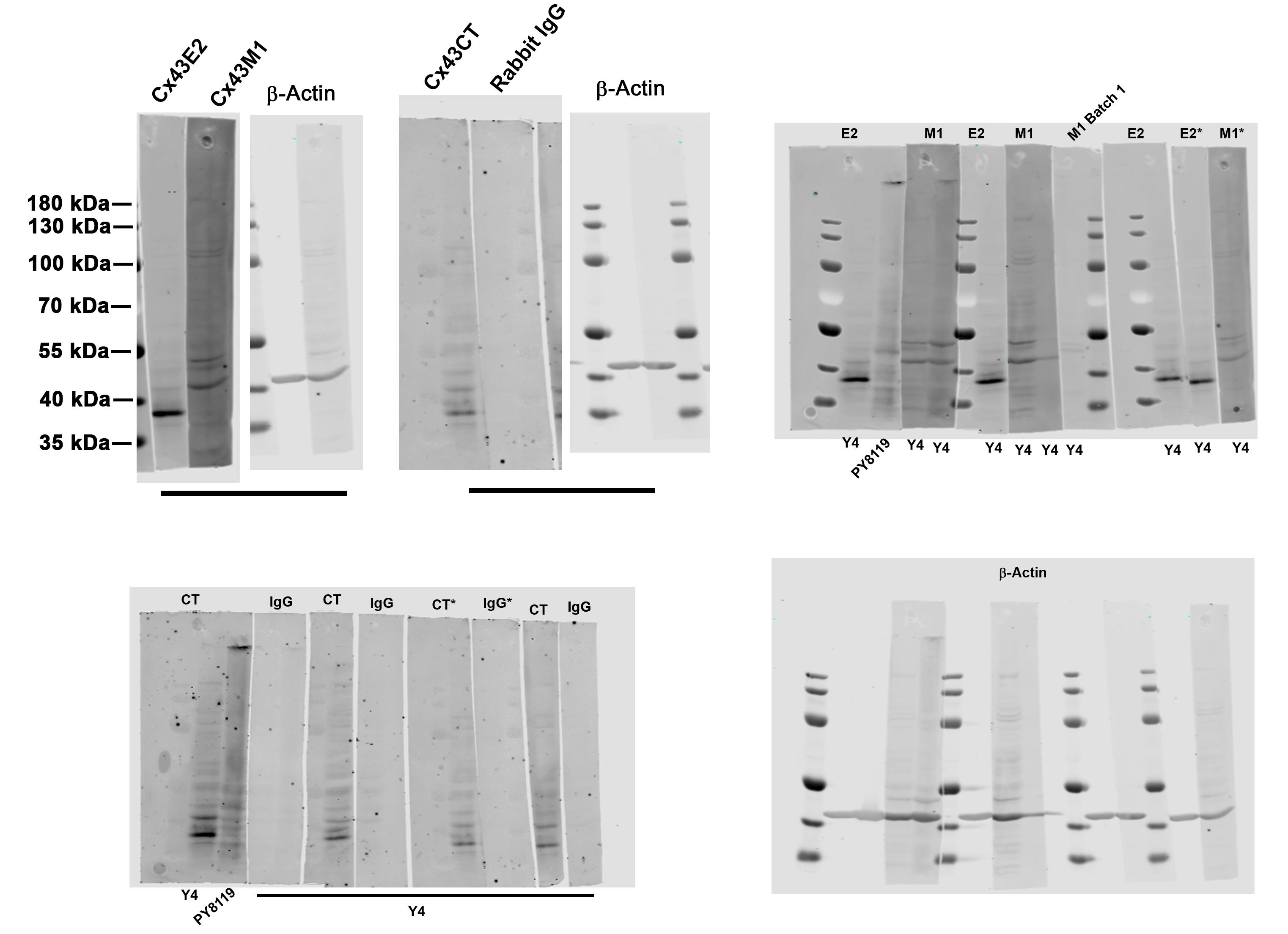

Supplement: Figure 5—figure supplement 1—source data 1. [file elife-74365-fig5-figsupp1-data1.zip › Figure 5-figure supplemental 1-source data 1.tif]
