# Supplementary material for: Connexin hemichannels with prostaglandin release in anabolic function of bone to mechanical loading
Source: eLife. 2022 Feb 8;11:e74365. doi: 10.7554/eLife.74365 (PMC8824479; doi:10.7554/eLife.74365)
Supplement: Supplementary file 1. [file elife-74365-supp1.docx]

**Supplementary file 1.** Sequences of the primers used for each gene used in this article

| Name | Sequence |
| --- | --- |
| *Sost*-F | CATCCCAGGGCTTGGAGAGTA |
| *Sost*-R | TGTCAGGAAGCGGGTGTAGT |
| *Ptgs2*-F | CCTTCTCCAACCTCTCCTACTA |
| *Ptgs2*-R | GGAAGCTCCTTATTTCCCTTCA |
| *Runx2*-F | CTCTGGCCTTCCTCTCTCAGTAA |
| *Runx2*-R | TAGGTAAAGGTGGCTGGGTAGT |
| *Bgalp2*-F | AGCAGGAGGGCAATAAGGTAGT |
| *Bgalp2*-R | TAGGCGGTCTTCAAGCCATACT |
| *Ctnnb1* -F | GACACCTCCCAAGTCCTTTATG |
| *Ctnnb1* -R | CTGAGCCCTAGTCATTGCATAC |
| *Dmp1*-F | CCCAGTTGCCAGATACCACAATAC |
| *Dmp1*-R | GCTGTCCGTGTGGTCACTATTT |
| *Gapdh*-F | CTTCAACAGCAACTCCCACTCTTC |
| *Gapdh*-R | TCTTACTCCTTGGAGGCCATGT |
